# A novel dynamic multicellular co-culture system for studying individual blood-brain barrier cell types in brain diseases and cytotoxicity testing

**DOI:** 10.1038/s41598-018-26480-8

**Published:** 2018-06-08

**Authors:** Patricia Miranda-Azpiazu, Stavros Panagiotou, Gin Jose, Sikha Saha

**Affiliations:** 1 Leeds Institute of Cardiovascular and Metabolic Medicine, Faculty of Medicine and Health, University of Leeds, Leeds, UK; 2 School of Chemical and Process Engineering, Faculty of Engineering, University of Leeds, Leeds, UK

## Abstract

Blood brain barrier (BBB) cells play key roles in the physiology and pathology of the central nervous system (CNS). BBB dysfunction is implicated in many neurodegenerative diseases, including Alzheimer’s disease (AD). The BBB consists of capillary endothelial cells, pericytes encircling the endothelium and surrounding astrocytes extending their processes towards it. Although there have been many attempts to develop *in vitro* BBB models, the complex interaction between these cell types makes it extremely difficult to determine their individual contribution to neurotoxicity *in vivo*. Thus, we developed and optimised an *in vitro* multicellular co-culture model within the Kirkstall Quasi Vivo System. The main aim was to determine the optimal environment to culture human brain primary endothelial cells, pericytes and astrocytes whilst maintaining cellular communication without formation of a barrier in order to assess the contribution of each cell type to the overall response. As a proof of concept for the present system, the effects of amyloid-beta 25-35 peptide (Aβ25-35), a hallmark of AD, were explored. This multicellular system will be a valuable tool for future studies on the specific roles of individual BBB cell type (while making connection with each other through medium) in CNS disorders as well as in cytotoxicity tests.

## Introduction

The blood brain barrier (BBB) is a specialised structure separating the central nervous system (CNS) from the peripheral blood circulation. It is crucial for maintaining the homeostasis of the brain microenvironment and prevention of entry of toxic substances into the CNS^1,2^.

The BBB consists of brain microvascular endothelial cells interconnected by tight junctions, which are one of the most important features of the BBB. Although brain endothelial cells are responsible for formation of tight junctions, both pericytes and astrocytes have also been shown to participate in their formation^3–7^, and thus are critical for maintaining normal BBB physiology and function as a barrier.

Despite the fact that several BBB barrier models have been created, most lack the ability to study individual BBB cell types separately, whilst maintaining communication between them. For instance, the function of pericytes in the BBB formation is still unknown, but has been described as essential to maintain BBB properties^4,6,8–17^. Although some BBB models include pericytes, these cells are usually not obtained from human brain. On the other hand, astrocytes are necessary to provide growth factors, nutrients and oxygen in the BBB^3,8,10,18–25^, now being widely used to improve the *in vitro* endothelial cell culture^2,3,20,26,27^. However, pericytes and astrocytes are not studied separately while communicating with each other and also with endothelial cells.

Comprehension of the factors that allow paracrine signalling when cells are not forming a barrier, but are able to communicate amongst them, could help in the design and improvement of future BBB models using human primary cells, identification of therapeutics targets for BBB integrity preservation as well as early detection of toxic effects over each specific cell type conforming the BBB.

BBB dysfunction has been linked to Alzheimer’s disease (AD)^28,29^. One of the pathological hallmarks of AD is extracellular deposition of senile plaques of amyloid β (Aβ) peptides in the brain, but the mechanisms by which Aβ peptide leads to AD are not yet fully understood. Different Aβ protein subtypes are known to cause inflammation and changes to BBB function. At high concentrations (nanomolar to micromolar), Aβ causes neurotoxicity and cell death^30^. Among the Aβ fragments studied so far, the Aβ 25-35(Aβ25-35), corresponds to the biologically active fragment of the full-length Aβ1-42 peptide that retains full toxicity^31^. A clear breakdown of the BBB barrier was demonstrated *in vivo* by Evans-blue extravasation in rat brain only 30 min after Aβ25-35 infusion into the right common carotid artery^32^. However, the mechanism of action of this peptide on each specific cell type shaping the BBB is still unknown.

Thus, in the present study, we set out to develop an *in vitro* multicellular system by culturing the human primary cell types, brain primary endothelial cells (HBECs), pericytes (HBVPs) and astrocytes (HAs) within the Kirkstall Quasi Vivo 500 system (QV500). This system allows multiple cell types to be cultured in interconnected chambers under flow whilst sharing the same culture medium. Although the different cell types are not in close contact, this model enables cell-cell communication through the sharing of the medium, resembling better physiological interactions when they are exposed to different compounds without formation of a true barrier. The main aims of the present study were i) to develop the best culture and maintenance conditions for these cell types (an improved culture medium, appropriate scaffolding systems and the optimal flow rate) in order to create a multicellullar co-culture flow system and ii) to check the feasibility of this multicellular system for toxicity screening on each cell type separately. As a proof of concept to achieve the second aim, we harnessed this system to explore the possible specific toxic effects of Aβ25-35 on brain endothelial cells, astrocytes and pericytes, while maintained cell-cell communication without formation of a true barrier.

## Results

### Determination of cell phenotypes by immunocytochemistry

**Figure 1:**
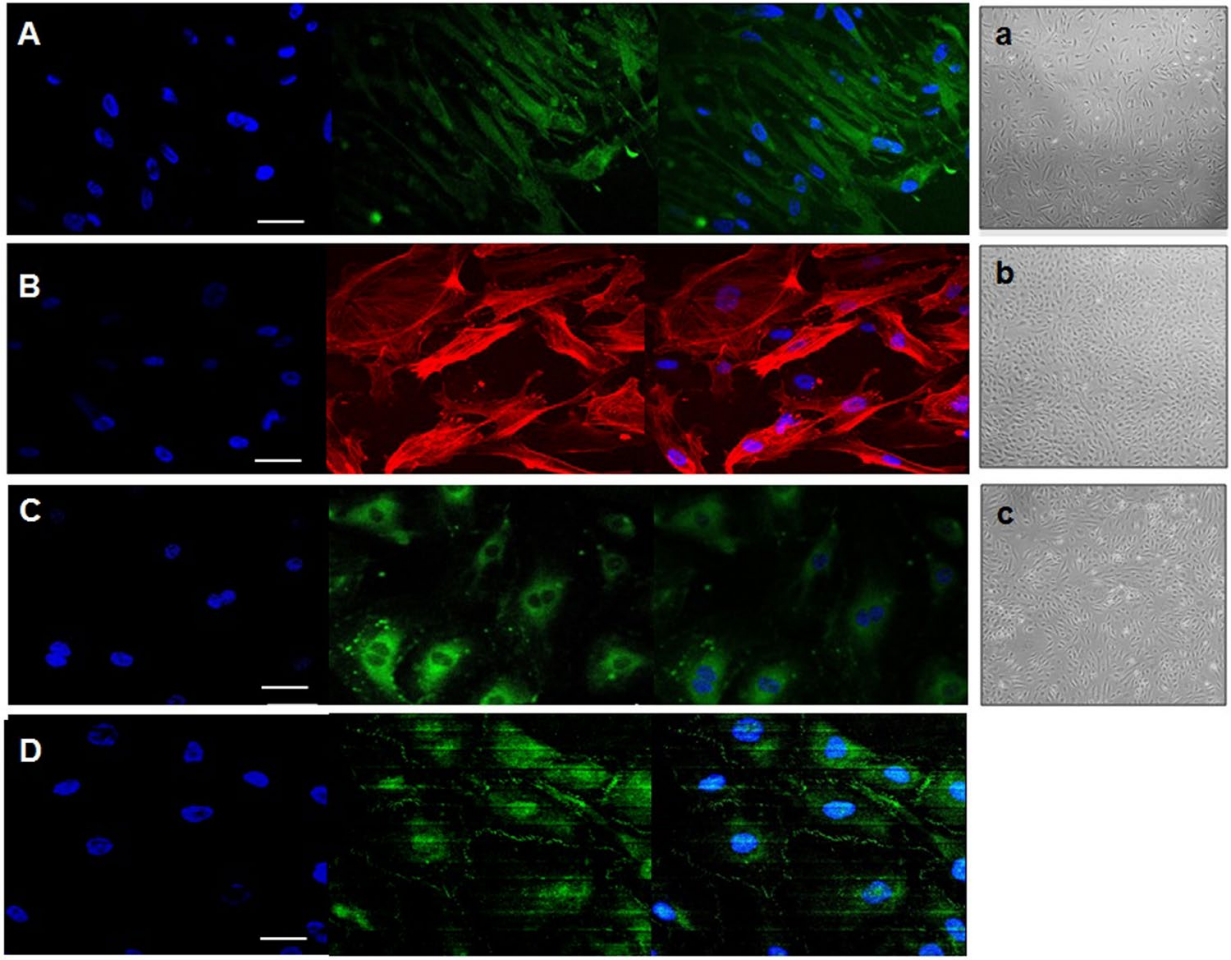
Representative immunocytochemistry image obtained using a Zeiss LSM700 or LSM 510 confocal microscope. (**A**) Atrocytes stained with GFAP, (**B**) pericytes stained with α-actin, (**C**) endothelial cells stained with CD31 and (**D**) endothelial cells forming tight junctions (*zonula occludens*, ZO1) (white arrows). Scale bar = 50 μm. In the right, representative images of the three human primary cell lines – astrocytes (a), pericytes (b) and endothelial cells (c) – cultured independently at early stages (passage 2) are shown. Images were taken from an optical microscope (Nikon Eclipse TS 100) at 20× augmentation.

To investigate if the human primary cells exhibited altered or expected phenotypes at early passages, specific antibodies, which have been used widely to confirm identity of these cell types were selected. As shown in Fig. 1, immunocytochemical studies showed that human astrocytes were able to selectively express glial fibrillary acidic protein (GFAP) (Fig. 1A), pericytes expressed α-actin fibres (Fig. 1B), and endothelial cells expressed CD31 (Fig. 1C), showing their expected morphology corresponding to one of the most characteristic features studied for each cell type. In addition, the ability of endothelial cells to form tight junctions under static conditions was confirmed by antibody labelling of the tight junction marker, *zonula occludens* (ZO1), thereby demonstrating they were able to express this marker even in the absence of flow (Fig. 1D).

### BBB Multicellular co-culture system

To construct a BBB multicellular co-culture system, we used HBECs, HBVPs and HAs. Firstly, we determined the best scaffolding and optimal medium for these cell types individually. The original media available for each cell type were improved and tested in order to find the best mix to culture and maintain successfully the three cell types under the same conditions. When the improved medium was established, flow was applied to each cell type individually and to the three cell types together. Control cells were cultured in parallel under identical conditions without flow. These experiments were carried out for the first time within the Kirkstall Quasi Vivo system. We have tried to characterize the optimal flow rate for each cell type and the best culture conditions in order to create the optimal environment to develop an *in vitro* dynamic BBB model using transwells in the future. The morphological phenotypes of the cell types cultured independently at early stages (passage 2) at low magnification levels (20x) and close to reaching confluence are shown in Fig. 1a–c.

### Determination of optimal flow rate

**Figure 2:**
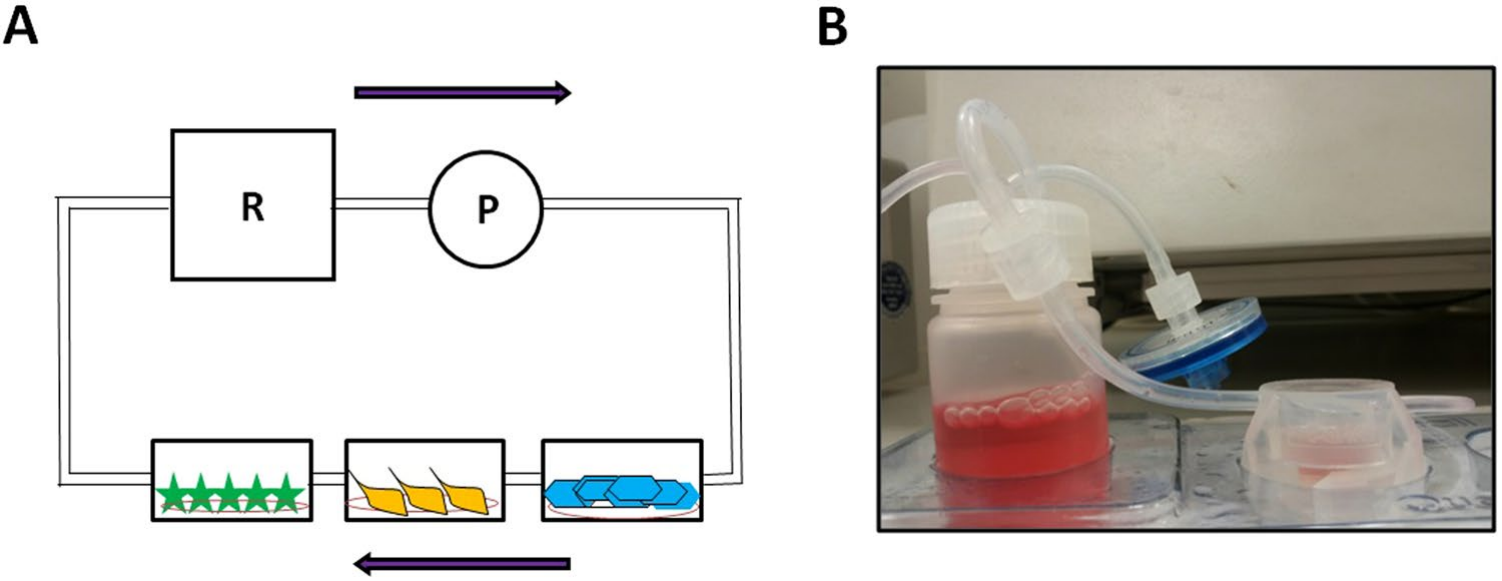
Schematic representation of Quasi-Vivo QV500 system (Kirkstall Ltd) (**A**) and a real image of QV500 system showing a single QV500 chamber connected to the reservoir containing cell culture media (**B**).

After the optimal medium was achieved and the expected features for each cell type confirmed, the effect of flow for each cell type compared to the static conditions was evaluated. To determine the highest flow rate that the cells could sustain, experiments with each cell type within the QV500 chambers (Fig. 2B) were carried out in triplicate and compared to the results obtained for each cell type under static conditions. Different flow rates were used to determine the optimum shear stress for all three cell types, up to 300 µl/min (0, 50, 100 and 300 µl/min). These values correspond to shear forces of 0, 2 × 10^−6^, 2.9 × 10^−6^ and 6.5 × 10^−6^ Pa, respectively. As expected, endothelial cells were able to survive at higher flow rates than 2.9 × 10^−6^ Pa, whereas astrocytes and pericytes died when the flow rate exceeded 2 × 10^−6^ Pa (data not shown). Thus, an optimum flow rate of 50 µl/min for growing and maintaining all three cell types was chosen for all subsequent experiments (Fig. 2A for schematic representation). This flow rate corresponds to wall shear stresses of 2 × 10^−6^ Pa (equivalent to 2 × 10^−5^ dynes cm^−2^) at the cell culture surface, and a flow speed of 2.6 × 10^−7^m/s. The ideal equilibrium was reached for all 3 cell types under the same conditions. This provided advantage of interconnection within all three cell types as well as the continuous flow of improved medium through the chambers, therefore maintaining a crucial homogeneous environment for all BBB elements while keeping them separate and maintaining their original individual features.

### Multicellular co-culture system using different combinations of cells

**Figure 3:**
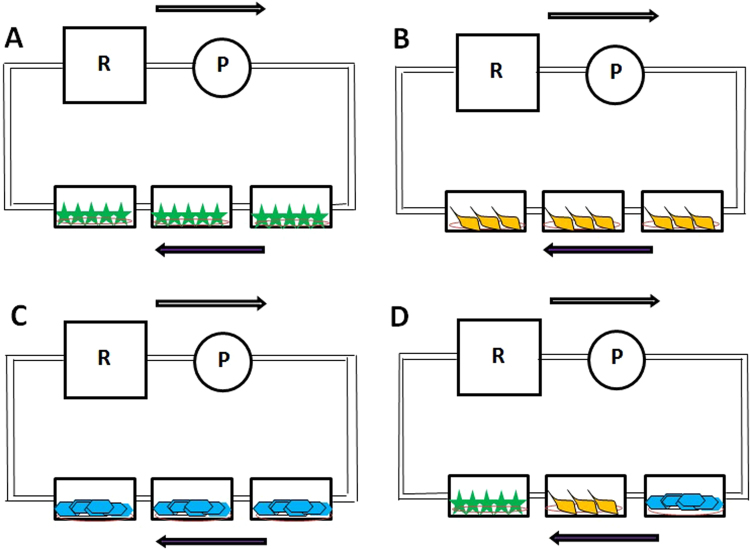
Schematic representation of the different combinations of cells cultured inside the QV500 chambers when using Kirkstall QV500 interconnected chambers. Each independent diagram shows the three cell types cultured alone in triplicate (**A**–**C**): astrocytes (green), pericytes (orange) and endothelial cells (blue), respectively), or together (**D**): astrocytes, pericytes and endothelial cells). T: Tank (media reservoir), R: Rotor (peristaltic pump). Arrows show the direction of the applied flow rate.

**Figure 4:**
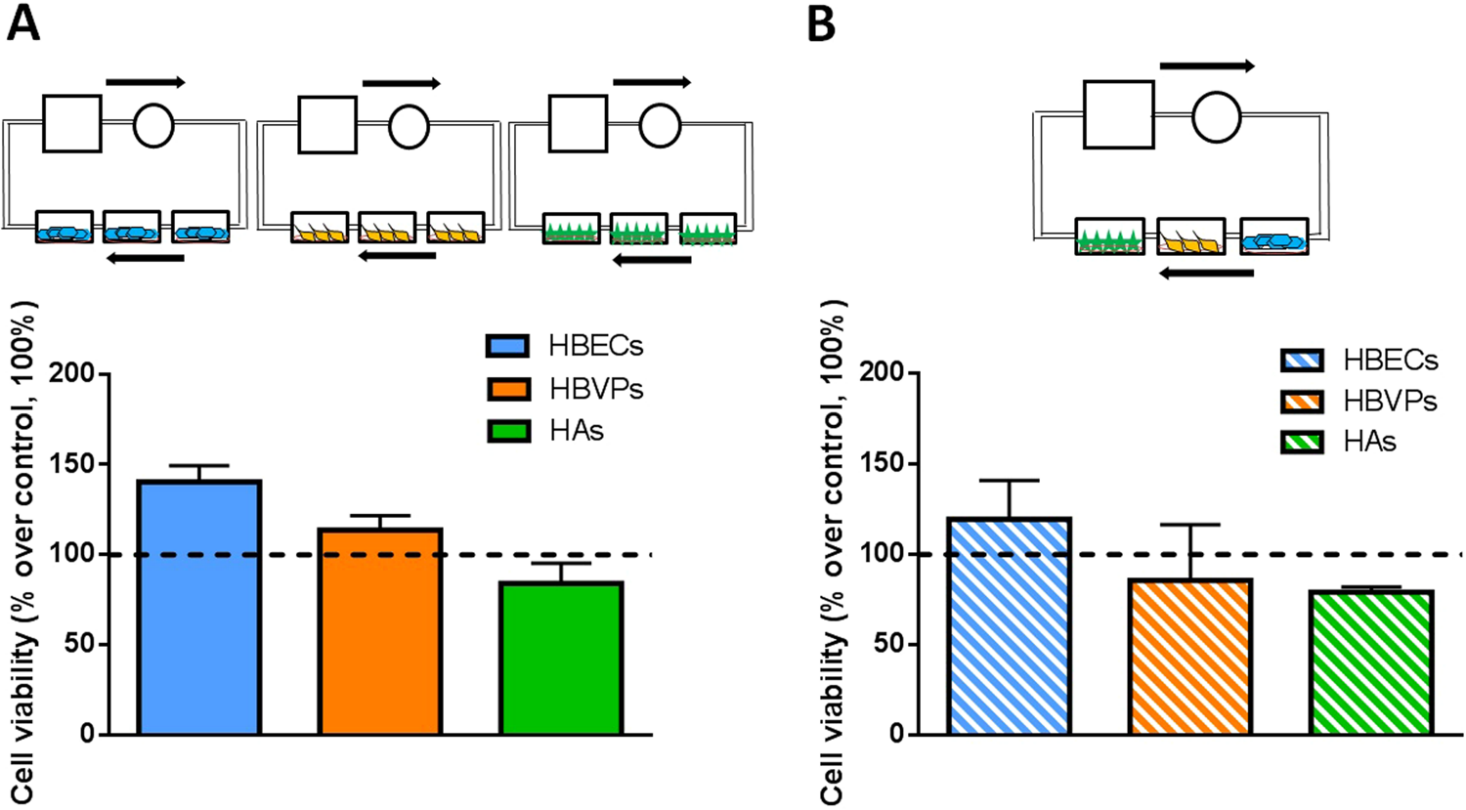
Cell viability measured by MTT assay comparing the three cell types under flow (50 µl/min) individually and in triplicate after 72 h inside the QV500 chambers (**A**) and the three cell types under flow (50 µl/min) together (one replicate of each cell time *per* experiment) (**B**). Results in both cases were normalized using the static conditions-control (black dotted line, 100%) from 3 to 8 independent experiments. No significant differences were observed.

In order to determine the importance of the culture of the 3 cell types alone or together, cells were cultured under flow separately (three replicates of each cell type inside the QV500 chambers, Fig. 3A–C) and one of each cell type inside every QV500 chamber (Fig. 3D). The aims of these experiments were to determine the importance of the co-culture of the cells together, the importance of the use of the improved medium and the effect of flow on cell viability. As shown in Fig. 4, no statistically significant differences were observed when comparing the cell viability using MTT assay for each cell type alone (A) and together (B) when using a 50 µl/min flow rate after 72 h inside the QV500 system in triplicate and compared to static conditions (represented as 100%). However, a tendency of a higher viability of HBECs was observed when they were cultured in triplicate (Fig. 4A, one-way ANOVA, p = 0.07, n = 3). In fact, analysis of HBECs cultured alone in triplicate showed a significant viability improvement after 72 h under flow (percentage increase over control of 40.5% ± 15.5) when compared to static conditions (100%) (Student’s t test, p = 0.045, n = 3). The pattern for all the cells studied was the same when culturing cells separately or together under the flow.

**Figure 5:**
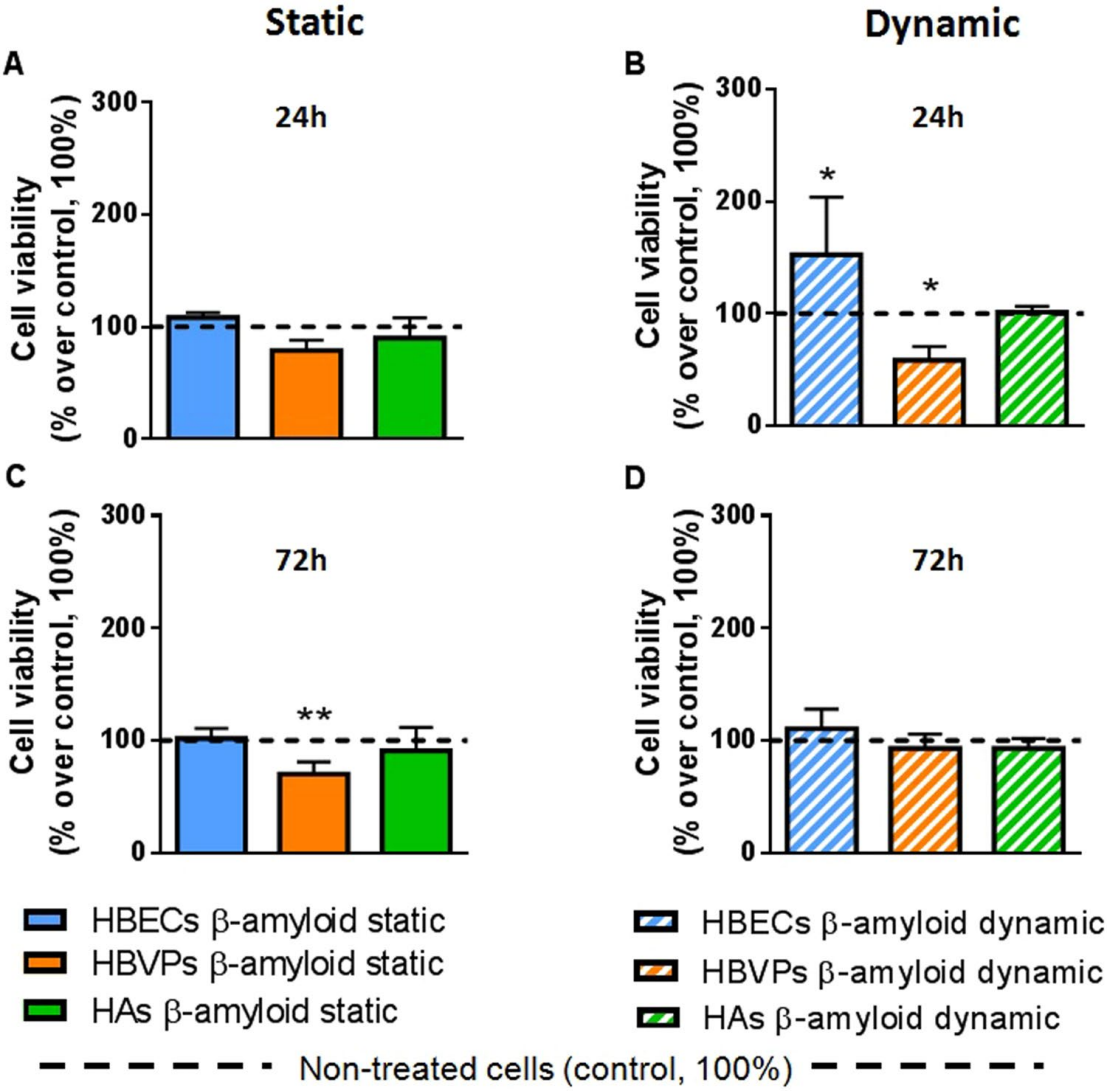
Cell viability measured by MTT assay comparing the three cell types under static and dynamic conditions in the presence and the absence of Aβ25-35. HBECs (blue bars), pericytes (orange bars) and astrocytes (green bars) where treated with Aβ25-35 (20 µM) in static conditions during (**A**) 24 h and (**B**) 72 h and under dynamic conditions (50 µl/min) inside the QV500 chambers during (**C**) 24 h and (**D**) 72 h and compared with control (absence of Aβ25-35 and represented as the black dotted line, 100%). Results are shown in percentage over control (100%) of 3 independent experiments. Significant differences in cell viability were obtained between Aβ25-35 treated and non treated cells when one-way ANOVA test (Tukey’s multiple comparison) was performed (*p < 0.05, **p < 0.01).

### Effects of Aβ25-35 on each cell type

Once the optimum cell culture and flow conditions had been established using QV500 chambers, the cells seeded in the coated coverslips, further experiments were performed exploring the effect of Aβ25-35 on each cell type to get a proof of concept of the multicellular co-culture system. The design, procedure and analysis were the same as for the flow rate characterization, using only 50 µl/min at different treatment times (24 and 72 h) and always feeding the cells with the improved media (in the presence and absence of Aβ25-35, 20 μM).

The same viability was maintained after 24 h treatment with Aβ25-35 when compared to the control (in the absence of Aβ25-35), showing no perceivable effect of the compound under static conditions (Fig. 5A). However, as it is shown in Fig. 5B, Aβ25-35 treatment affected different cell lines in different ways under dynamic conditions when compared with the non-treated controls (one-way ANOVA, F = 5.151, p = 0.0008, n = 3). In fact, Aβ25-35 affected the viability of pericytes after only 24 h of exposure in a negative way (58.5% ± 12.1), prompting a significant 41.5% decrease in viability (Fig. 5B), compared with control, expressed as 100% (One-way ANOVA, Post Hoc Tukey, p = 0.02, n = 4). Endothelial cells showed an increase in cell viability (152.6% ± 89.0) when compared with control, showing a significant 52.6% improvement (One-way ANOVA, Post Hoc Tukey, p = 0.02, n = 3). Under these conditions, astrocytes did not suffer any significant effect compared to control (101.5% ± 9.1; One-way ANOVA, Post Hoc Tukey, p = 0.8, n = 3).

Notably, after 72 h of Aβ25-35 treatment an effect on the viability of pericytes was only observed under static conditions (viability of 70.2% ± 23.9; One-way ANOVA, Tukey’s multiple comparison test, n = 3, p = 0.0029), whereas no effect of Aβ25-35 was shown in any other cells regardless of application of shear stress (101.9% ± 2 for HBECs and 90.9% ± 41.3 for HAs, Fig. 5C,D). No statistically significant changes were obtained either when controls under the flow were compared to static controls (data not shown), as expected due to the aforementioned flow rate characterization (Fig. 4).

## Discussion

Investigation of the dynamic metabolism and numerous roles of each of the diverse BBB components necessitates development of a suitable *in vitro* system capable of determining the role of individual cell types separately to understand the contribution of each cell type to the overall response. During the last decade, there has been increasing interest in determining the basic design requirements for generating physiologically relevant *in vitro* systems^33^.

In the present study, we have constructed a simple multicellular co-culture system under flow within Kirkstall Quasi Vivo interconnected chambers using human primary brain endothelial cells, human brain vascular pericytes and human brain astrocytes. The scaffolding system for each cell type and the optimum flow rate to culture and maintain the three cell types under the same conditions were established as a priority aim. The different flow rates applied showed that 2 × 10^−5^ dynes cm^−2^ was the optimum shear stress able to maintain the viability of all three cell types closest to that observed under static conditions. The BBB tight junction formation was not assessed by measuring transendothelial electrical resistance (TEER) of endothelial cells as we did not create a barrier model but a multicellular co-culture system for toxicological studies. However, we have determined the tight junction expressed using immunocytochemistry in order to ensure that the human brain primary endothelial cells chosen for the study maintained their original features, and that they had not changed their morphology during passaging process.

The multi-compartmental modular Kirkstall Quasi Vivo system was chosen due to the multiple advantages of co-culturing different cell types in monolayers, thereby improving the cell function^34^. This milli-scaled system maintains the same protocols used in traditional static multi-wells as it has shape and dimensions similar to those of a classic 24-multiwell cell culture plate (13 mm diameter and 11 mm height)^33^. It allows chambers to be connected in series or parallel as required, and the main features of the system are the absence of air bubbles, high oxygen transport through convection, and the possibility of connecting additional chambers^35^. One of the main disadvantages of milli-scale methods is the difficulty to analyse the contribution of each cell type to the co-culture function. The design of the Kirkstall Quasi Vivo independent chambers abolish this disadvantage. This system has been previously used to create other organ-flow systems growing different cell types, and studied in detail by several authors^33,35–39^. The Quasi Vivo system has been shown to have low shear stress, a short time scale for test compound distribution, and sufficient oxygen availability at higher flow rates^40^.

Despite the fact that other systems have been developed, this is believed to be the first example of a multicellular system using BBB cells without forming a barrier, thereby allowing the study of each cell type separately. Other models have been published, but their format and purpose are completely different. For instance, the model recently designed and developed by Brown and collaborators^22^ corresponds to a microfluidic system on a chip, which is able to determine the passage of different compounds through BBB model. Although these micro and nano-scaled systems are fashionable and may mimic physiological interactions between cells, these devices remain a niche research tool. As explained by Mattei and collegues^33^, they are not representative of a tissue/organ and cannot meaningfully predict *in vivo* physiology or pathophysiology. Our system allows use of different cells on each experiment and the option to observe the individual effect of compounds over each cell type. This allows pre-screening of different drugs, nanoparticles or the molecule of interest in a more realistic environment. The aim of this study was not to develop a barrier but to investigate the effect of different compounds or conditions over each specific cell type.

It is well known that astrocytes are not supposed to suffer shear stress in the brain. However, we aimed to evaluate their possible change in terms of viability when they were co-cultured under interstitial flow-like conditions, as has been proposed in the literature^41,42^. Although endothelial cell viability under flow was not highly significant (p = 0.04) compared to the static conditions, it was clearly improved (higher than a 53% increase of the viability), whereas astrocytes and pericytes remained unchanged. The same pattern was observed when a single cell type was placed inside Quasi Vivo chambers (Fig. 4A) and when the three cell types were placed in connecting chambers (Fig. 4B). This suggests that the system is working as expected, and that astrocytes are also able to resist shear stress. This fact allowed us to co-culture endothelial cells, astrocytes and pericytes together under the same conditions. It is important to point out that astrocytes would not be under shear effects in a BBB model even though they are exposed to interstitial flow. However, it was necessary in this study in order to enable communication between cell types without a direct contact.

There is increasing evidence that AD induces complex changes in barrier functions^43^, but the individual contribution of these cell types in the disease mechanisms is not clear. Thus, one of the secondary aims of the present work was to explore the possible effects of Aβ25-35 fragment on BBB individual cell types under dynamic and static conditions as a proof of concept for this system. Aβ25-35 is the shortest biologically active fragment of full-length Aβ (1-42) that retains full toxicity^31^. Aβ25-35 was chosen as a proof of concept for this multicellular co-culture system because this peptide has been shown to produce membrane perforation, calcium increase and synaptotoxicity, and this fragment exhibited neurotrophic and neurotoxic activities similar to those of the (1-40) peptide^44^. The differences in terms of toxicity for different Aβ fragments have been already studied by other groups^45^.

Several studies have reported the cytotoxic effects of Aβ25-35 in different cell lines^31,32,46,47^. In fact, a recent study showed how cell viability was significantly decreased after exposure to 5, 10, 20 and 30 μM Aβ25-35 for 24 h, and reported that the half maximal inhibitory concentration (IC_50_) was 20 μM when using SH-SY5Y cells^46^. Thus, the chosen Aβ25-35 concentration for our preliminary experiments was 20 μM.

In the present study, pericytes were affected by Aβ25-35 exposure after only 24 h treatment under flow, whereas endothelial cells and astrocytes were not affected in the early stages of Aβ25-35 toxicity (Fig. 5B). This fact is in agreement with previous studies where cultured human brain pericytes alone were used to study the mechanisms of microvascular amyloid formation and cytotoxic effects of Aβ on perivascular cells^48,49^. This effect was not observed in a significant manner under static conditions (Fig. 5A). The degenerative effect that pericytes showed after 24 h treatment under a shear stress was observed after 72 h under static conditions (Fig. 5C), an effect that was completely abolished under flow (Fig. 5D). This result might suggest that an initial flow of 2 × 10^−5^ dynes cm^−2^ is able to promote great differences following a specific treatment in early stages. It also suggests the possible compensatory effects that could be driven by co-culturing the three cell types together when a flow is applied, a fact that cannot be observed when cells were cultured in static terms (even though the same conditions were followed except the flow). In fact, after 72 h of co-culture under dynamic conditions, no negative or positive significant effect over any of the three cell type’s viability was observed, suggesting that both toxic and compensatory effects could be different when applying shear stress, and implying the possible importance of these shear forces over this system containing a multicellular co-culture model. Moreover, it has been shown how the effect of Aβ25-35 is cell –type specific, in agreement with a previous report^46^.

In the present study, we did not explore the complex mechanism of AD pathogenesis which is beyond the scope this study. Although the mechanisms of Aβ25-35 are not well understood, previous studies have reported that it exerts the toxic effects by production of inflammatory cytokines, reducing glucose metabolism and inhibiting cytoprotective proteins^50–52^. We would expect that the concentration of paracrine signalling molecules close to the cells is affected by the flow rate, media volumes, the diffusion constant, cellular production and uptake rates, molecular half-life and adsorption onto the walls of the device. In the case of hydrophilic molecules such as Aβ25-35 or cytokines, the latter factor is negligible as PDMS preferentially adsorbs small hydrophobic moieties^53^. Besides the fluid dynamics of the system, which is quite well-characterised^33,34^, to build a full picture of the signalling environment requires a combination of cellular output of signalling molecules including the response by the cells to the change to this environment. The total volume within the system will dilute concentrations; however typical signalling molecules such as cytokines have high production rates. It has been reported a production rate of 4 molecules per cell/s^54^, half-lifetimes of the order of 30 minutes) and very low equilibrium reaction constants (10 pM)^55^. Although beyond the scope of this investigation, understanding flow systems more broadly in this type of framework combined with experimental models could help build a more detailed understanding of cellular behaviour.

Further research is needed in this field to explore the exact mechanism of action of Aβ25-35 on pericytes and determine if the observed decrease in cell viability could directly affect the permeability of the BBB and, in turn leading to BBB leakage (using a new BBB model with the formation of a barrier).

In conclusion, this multicellular co-culture system could become a useful tool for toxicity screening to evaluate the specific effects of neurotoxic, neuroinflammatory and neuroinfectious agents on each cell type separately and would provide improved conditions to develop a BBB dynamic model using human primary cells in the future.

## Material and Methods

### Immunocytochemistry

Briefly, the coverslips containing each specific cell type were washed twice in 0.1 M phosphate buffered saline (PBS, pH 7.6) and fixed with fresh 4% paraformaldehyde (PFA) for 10 minutes. The PFA was discarded and the coverslips were washed 3 times in PBS for 5 minutes each. The coverslips were placed into a 12- or 24-well plate and were re-suspended in 10% horse serum for 1 hour at room temperature. Primary antibody incubation was carried out for 2 hours at room temperature or overnight at 4 °C with gentle agitation. The primary antibodies used to stain HAs, and HBVPs were Glial Fibrillary Acidic Protein (GFAP) and α-actin respectively (both from Santa Cruz Biotechnology, UK, catalogue numbers sc-33673 and sc-32251, respectively). HBECs were identified by using primary antibodies against CD31 and *Zonula Occludens* (Rb anti ZO-1), both from Thermo Fisher, catalogue numbers PA5 16031 and 40–2200, respectively). All antibodies were used in a 1:100 dilution except HBVPs and CD31, which were used in 1:300 and 1:40 dilutions, respectively. After incubation with the primary antibody, the samples were washed in PBS for 3 × 5 min. The corresponding secondary antibody conjugated to Alexa Fluor (1:1000) or Cy3 (1:500) was added to the cells for 1 hour at room temperature with gentle agitation. Cells were washed in PBS for 3 × 5 min, and the coverslips were inverted onto a drop of mounting medium containing DAPI (Vectashield antifade mounting medium with DAPI, Vector Laboratories, UK) on a microscope slide, and stored at 4 °C. The immunostained cells were viewed under confocal (Zeiss LSM 700 inverted or Zeiss LSM 510 upright) or fluorescence (Zeiss Axiovision) microscopes using appropriate excitations for each fluorophore. All images were imported into ImageJ-Win64 program for minor adjustment of brightness and contrast, resizing or cropping and assembling into figures. After lettering, the layers were merged and the images were saved as TIFF files using Microsoft Office PowerPoint.

### Human brain primary cells (astrocytes, pericytes and endothelial cells) culture

Human primary cells were purchased from available commercial firms, which are committed to the highest ethical and legal standards and approvals. Endothelial cells (CSC-C1503, passage 0) were obtained from Creative Bioarray (Creative Dynamics, Inc, UK), whereas astrocytes (1800-5) and pericytes (1200) were both obtained from ScienCell (UK), purchased through Caltag Medsystems (UK).

In all cases, cells were cultured in T75 flasks until 85–90% confluence (Fig. 1) to avoid possible cell differentiation or inhibition processes. Human brain primary endothelial cells (HBECs) were cultured following the protocols of Patabendige and Abbott (2014) with minor modifications. Endothelial Cell Growth Medium MV2 (Promocell, UK) was used to feed HBECs every 2 days until the cells reached ~90% confluency. Astrocyte medium from primary human astrocyte (HA) culture (conditioned medium) was combined with endothelial cell medium as previously described by other groups^56^ in a 1/3 proportion. The medium was filtered using Millipore Express (PES) Membrane (pore size 0.22 μm, diameter 33 mm, sterile, γ-irradiated) and stored at −80 °C until use. HBECs and Human Brain Vascular Pericytes (HBVPs) conditioned media were also collected and stored in exactly the same way as described for astrocytes for further experiments of co-culture with “improved medium”.

Passage of cells was carried out by standard trypsinisation (SigmaAldrich, UK). After centrifugation, the pellet was re-suspended and cells counted using a haemocytometer. All flasks, plates and coverslips were coated beforehand using collagen type I from rat tail (Sigma Aldrich, UK), following the protocol described elsewhere^2^. These cells were able to maintain their original phenotype and grow fast up to passage seven. However, cells were only used up to passage 5 in the present study. Both HAs and HBVPs were cultured with Dulbecco’s modified Eagle’s Medium/Ham’s Nutrient mixture F12 (DMEM) (Sigma Aldrich, UK), supplemented with 10% sterile filtered foetal bovine serum (FBS) of South American Origin (Labtech.com), 1% antibiotics (Penicillin – Streptomycin) and 1% L-Glutamine (Sigma Aldrich, UK).

The improved medium consisted of filtered conditioned medium obtained from all cell types and mixed with fresh medium. The final improved medium also contained a higher volume of fresh media (60%) than filtered media (40%). The 40% of filtered media consisted in equal parts of each cell line’s conditioned media. Final volumes were variable depending on the experiment, but this proportion was always strictly maintained. Cells were supplemented with fresh improved medium every 3–4 days, depending on the requirements, until confluence was achieved. The trypsinisation process was carried out using the method as described previously.

Flasks, plates and coverslips used to culture HAs and HBVPs were coated with Poly-d-lysine hydrobromide (Sigma Aldrich, UK), following the steps described elsewhere^2^.

These cells were able to maintain their original phenotype and grow fast up to high number of passage, although were used for experiments at early stages (up to passage 5).

### Co-culture of astrocytes, pericytes and endothelial cells using Kirkstall Quasi vivo (QV500) system

After connecting all the components of the QV500 system as explained by the manufacturer (http://www.kirkstall.com/why-quasi-vivo/), the system was sterilised with 70% ethanol by allowing the alcohol to pass through the system at a flow rate of 200 µl/min for 24 hours. The solvent was removed, the components dried and PBS was passed through the system to remove any ethanol residues. Finally, the system was flushed with 1% antibiotics (Penicillin – Streptomycin) in PBS for at least 24 hours to ensure sterility prior to the addition of cells. The QV500 system was assembled inside a class II cabinet and the coverslips carrying cells were placed in separate chambers (Fig. 2). Then, 1 ml of medium was added to each chamber with the cells facing up. The tank providing the cells with nutrients was supplemented with 30 ml of improved medium (Fig. 2B).

Combinations of cells were carried out to investigate the effect of a serial setup. Three replicates of a single cell type, (Fig. 3A–C) or the 3 cell types together (Fig. 3D) were cultured in order to place them inside QV500 chambers (Fig. 2B) the previous day of the experiment. In this system, the three QV500 chambers are interconnected and sharing the medium coming from the reservoir, which is gently pumped according to the desired flow rate impulse by the peristaltic pump (Fig. 2A for scheme). In order to increase the reproducibility of the results, the density of the cells used was always 105 per experiment for every cell type. This density was enough to form a monolayer of cells covering completely the coverslip after 24 h of culture. Cells were seeded using the correspondent scaffolding method (depending on the cell type) following the aforementioned protocol and optimized medium, and were placed in the incubator overnight to ensure the cells had successfully attached to the coverslips. The following day, cells were ready for the experiment inside the QV500 chambers. Coverslips containing cells in triplicate (Fig. 3A–C) or one of each cell type (Fig. 3D) were placed facing upwards, and flow was maintained for 72 h. Control and flow-treated cells were kept in the same incubator for the duration of the experiment. A flow rate ranging from 0–300 µl/min (0–6.5 × 10^−6^ Pa) was applied to the system. The calculations used to obtain shear and flow speed values are described in equations 1 and 2, respectively^19^.

1 $${\rm{Shear}}\,({\rm{Pa}})={\rm{Flow}}\,(\mu {\rm{L}}/\min )\times 1.8\times {10}^{-8}+1.1\times {10}^{-6}$$

2 $${\rm{Flow}}\,{\rm{Speed}}\,({\rm{m}}/{\rm{s}})={\rm{flow}}\,(\mu {\rm{L}}/\min )\times 2.6\times {10}^{-9}+1.3\times {10}^{-7}$$

The QV500 system with cells under a constant flow was placed in the incubator. Cells were maintained inside the model under flow for 72 hours. As a viability control, the model was replicated in static conditions using a conventional 12 well plate, and placed in the same incubator at the same time. The impact of flow on cell viability was assessed by an MTT (3-(4,5-dimethylthiazol-2-yl)-2,5-diphenyltetrazolium bromide assay.

### MTT cell viability assay

Static experiments were carried out without flow by culturing the cells inside 12-well plates using the same protocol. Cells were incubated in the presence and absence of soluble Aβ25-35 (20 μmol/L) for 24 and 72 h assessing the possible effect of a specific flow rate (50 µl/min, 2 × 10^−5^ dynes cm^−2^). After the required incubation time, all the cells in the coverslips were placed in 12-well plates and washed twice with PBS. MTT assay was carried out by adding 1 mg/ml solution of MTT (Sigma M5655) in DMEM/F-12, HEPES, no phenol red from Gibco (catalog number: 11039021) and adding 500 µl of the solution into each of the wells on the coverslips. Cells were incubated for 4 hours at 37 °C protected from light. Then, 350 µl of isopropanol was added to the cells with gentle pipetting up and down and shaking at 400 rpm for 20 mins to dissolve the crystals formed. Finally, the liquid was transferred into a 96 well plate in triplicate for each condition and the optical density (OD) was measured on an IMark absorbance microplate reader (Bio-Rad, UK) with a test wavelength of 570 nm, using isopropanol as blank.

### Statistical analysis

All data are presented as mean ± SD of 3–6 independent experiments, being normally performed in triplicate. Data were analyzed by one-way ANOVA, with differences between groups assessed by Tukey’s post hoc tests. All graphs and analysis were performed using GraphPad Prism v6 software (GraphPad, San Diego, California). Statistical significance was determined when P < 0.05.
